# Involuntary Attention Restoration During Exposure to Mobile-Based 360° Virtual Nature in Healthy Adults With Different Levels of Restorative Experience: Event-Related Potential Study

**DOI:** 10.2196/11152

**Published:** 2018-11-30

**Authors:** Kyungmi Chung, Daeho Lee, Jin Young Park

**Affiliations:** 1 Department of Psychiatry Yonsei University College of Medicine, Gangnam Severance Hospital Seoul Republic of Korea; 2 Institute of Behavioral Science in Medicine Yonsei University College of Medicine Seoul Republic of Korea; 3 Department of Interaction Science Sungkyunkwan University Seoul Republic of Korea

**Keywords:** smartphone, virtual reality, attention, surveys and questionnaires, electroencephalography, evoked potentials

## Abstract

**Background:**

With the global trend of urbanization, there are increasing reports of a possible association between decreased exposure to nature and increased occurrence of mental disorders. New 360° virtual reality (VR) technology using smartphones and portable VR glasses can overcome spatial and temporal limitations to help people deal with mental fatigue in everyday life.

**Objective:**

On the basis of attention restoration theory (ART), this study aimed to investigate whether the amplitude of the mismatch negativity (MMN)/P3a complex could act as an event-related potential (ERP) biomarker of involuntary attention restoration during exposure to 360° virtual nature in healthy young adults with different levels of restorative VR experience.

**Methods:**

A total of 40 healthy adults completed prequestionnaires on demographics and simulator sickness and postquestionnaires on simulator sickness and perceived restorativeness before and after exposure to virtual nature, respectively. During the VR exposure, brain activity was measured by electroencephalography as participants were asked to conduct a 2-tone passive auditory oddball task.

**Results:**

The amplitude and latency of the MMN/P3a complex were compared between individuals reporting a highly restorative experience and those reporting a less restorative experience. Although viewing a virtual nature environment, the high restorative group (N=19) exhibited significantly reduced P3a amplitudes compared with the low restorative group (N=20; *t*_38_=2.57; *P*=.02; *d*=0.59). Particularly, a moderate but significant negative correlation was found between the self-reported restorativeness scores and the P3a amplitudes at the fronto-central region (*r*=−.38; *P*=.02). However, the latency of the MMN/P3a complex did not significantly differ between the 2 groups (auditory mismatch negativity: *t*_38_=−1.47; *P*=.15 and P3a: *t*_38_=−0.31; *P*=.76).

**Conclusions:**

Considering individuals’ restorative experience, the amplitude of the fronto-central MMN/P3a complex can potentially be employed as a distinct ERP component of interest in involuntary attention restoration during virtual nature experience in healthy young adults. The findings for the 360° virtual nature experience seem to be consistent with those of previous ERP studies on the effects of meditation practice. This study extends the findings of previous ART and ERP studies of real-world meditation, restoration, and mental fatigue management into the virtual world created by mobile phone–based VR glasses and 360° video content.

## Introduction

### Background

Although some people prefer to enjoy attention-drawing sport or entertainment events to recover from mental fatigue, why do others prefer to walk in or just be exposed to natural environments and why is nature so well known for its restorative effects? From the perspective of the attention restoration theory (ART) [1], exposure to nature serves as a vital ingredient in healthy human functioning, contributing to the replenishment of depleted attentional resources. Involuntary attention, also called *fascination*, is a central component of a restorative experience. Although involuntary attention requires no effort and is resistant to fatigue, directed or focused attention requires a capacity for concentration that can become depleted by taxing task demands, leading to stress responses or impaired performance [1-3]. Individuals can rapidly restore depleted attentional resources by using involuntary attention and allowing directed attention to be in a resting state [4]. Fascination ranges along a continuum from *soft* to *hard* [1]. Soft-fascinating activities (eg, walking in a park or seeing a sunset) are sufficiently distracting to hold one’s attention, but they leave room for mental reflection and are aesthetically pleasing, which helps to offset the pain that accompanies reflection on serious matters. In contrast, hard-fascinating activities (eg, sports or entertainment) fill the mind and rivet one’s attention in an all-consuming fashion, leaving little or no room for mental reflection [1,5]. A stream of hard-fascinating environmental events is considered as an incoherent collection of impressions and not a restorative experience [1]. Hence, a peaceful and moderate nature experience coupled with aesthetic pleasure fosters a fuller, more deeply restorative experience than a sports or entertainment experience does.

Despite the expected restorative effect of interaction with nature, it is difficult for modern people to deal with mental fatigue at the right time because of increasing urbanization as well as spatial, temporal, and social constraints. Prolonged mental effort results in directed attention fatigue [1]. Coincident with the global trend of urbanization, there is increasing evidence of a possible link between decreased exposure to nature and increased occurrence of mental disorders [6-8]. Direct contact with nature, such as walking in natural environments [9-11] and bringing plants and flowers into residential and office environments [12], leads to recovery from attentional fatigue and improvement in cognitive performance. In addition, people who suffer from directed attention fatigue can benefit from indirect contact with nature such as viewing nature through a window [13] or viewing videos or pictures of natural environments [14,15]. Regardless of whether the contact with nature is direct or indirect, it is important for people to have easy access to it so that they can reflect on their everyday life and restore their depleted attention in a timely manner. In that context, virtual reality (VR) technology can help to overcome possible constraints on access to nature; thus, offering a promising therapeutic alternative for individuals who are vulnerable to cognitive overload and are at a higher risk of mental illness.

**Figure 1:**
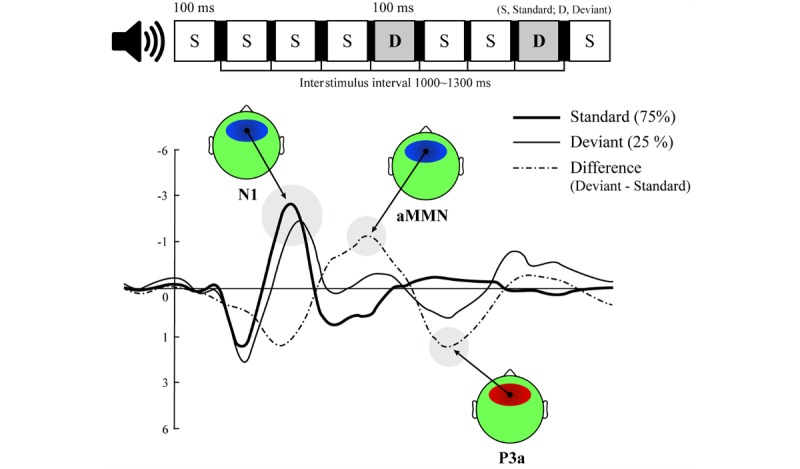
Illustration of expected event-related potential components (N1 and mismatch negativity/P3a complex) and their grand-averaged waveforms and topographic maps at the same region of interest (FCz electrode) evoked by a two-tone passive auditory oddball paradigm. aMMN: auditory mismatch negativity.

In recent ART studies, the event-related potential (ERP) method, which uses ERP components as scalp-recorded voltage changes that appear as a series of positive and negative peaks varying in polarity, amplitude, and duration and reflecting a specific neural or psychological process, has yet to be fully implemented to examine the effect of contact with real nature or even virtual nature. Traditional ART studies measure the restorative effects of natural environments on voluntary attention not only on the basis of subjective, self-reported responses such as the Perceived Restorativeness Scale (PRS) [16] but also on the basis of objective, behavioral responses to cognitive tests, particularly immediately after the nature exposure. Compared with those widely used methods, the ERP method bears the advantages of (1) measuring subjective and objective neuroelectrophysiological responses simultaneously during or after the performance of given tasks and (2) exploring the time courses of both early preattentive and late-attentive sensory processing using topographic and principal component analyses. This study focuses on a specific ERP component called mismatch negativity (MMN)/P3a complex, which is observed at fronto-central sites as a combination of the 2 different preattentive ERP components: MMN and P3a (see Figure 1). The negative-going MMN component (usually peaking at approximately 150-250 ms from the onset of the deviant stimuli in a passive auditory oddball paradigm) is followed by the positive-going P3a component (peaking at approximately 220-280 ms), reflecting a subsequent attention-orienting (or shifting) process as the neurophysiological *handover* from the MMN to the P3a [17-19]. Particularly, utilizing the MMN/P3a complex elicited in the absence of attention is appropriate to investigate the restorative effect of exposure to 360° VR nature on depleted involuntary attention, in that people can simultaneously perform a VR-based visual task in a passive auditory oddball paradigm, without intentionally regulating attention. On the basis of its capability for the early detection of sensory stimuli, the ERP method may provide people vulnerable to repetitive stressful events and cognitive overload with the possibility of early intervention with 360° VR exposure therapy and also a way to systematically evaluate the restorative effect of the therapy.

According to ERP findings during meditation, the mean auditory MMN (aMMN) amplitude of long-term mediators was larger than that of nonmediators, indicating that auditory fatigue or cognitive overload tended to reduce the aMMN amplitude [20]. Cahn and Polich [21] revealed that participants who reported more hours of daily meditation practice produced the strongest meditation-induced decrease in P3a amplitude. Given that the P3a reflects frontal cortical activity elicited by engagement of the focal attentional system [22], the reduced P3a amplitude may reflect the disengagement of attentional networks from stimulus-driven activation during meditation, which meets the goal of meditation to decrease brain reactivity to attention-demanding stimuli and evaluative cognitive processing.

Unlike the P3a, the aMMN is less likely to decrease significantly in amplitude with restorative activities because the aMMN, which is independent of attention [23], is not affected by the nature of the visual content and the immersiveness of visual tasks (from traditional reading task to VR tasks) [24]. In fact, the modulation of ERP amplitudes during meditative or restorative activities remains unclear because of a lack of investigations in applied ERP research settings. Furthermore, the aMMN and P3a have been investigated only separately, and previous ERP studies on meditation employed inconsistent experimental protocols.

### Objectives

The purpose of this study was to demonstrate the potential role of the amplitude of the MMN/P3a complex [17-19] in reflecting involuntary attention restoration during exposure to restorative nature virtual environments (VEs) in healthy young adults with different levels of restorative experience. ERP amplitudes during exposure to 360° virtual nature might be expected to exhibit patterns similar to those observed during meditative activities [20,21]. We hypothesize that compared with people who have a low level of restorative experience, those who have a high level of restorative experience will show a significant reduction in P3a amplitude but not in aMMN amplitude because of the preattentive nature of the aMMN in both the real world [23] and the virtual world [24]. We further hypothesize that there will be a negative correlation between self-reported PRS scores and P3a amplitudes.

## Methods

### Participants

A total of 40 healthy volunteers (22 males and 18 females) aged from 19 to 36 years (mean 23.78 [SE 0.56]) were recruited in the following 2 ways: (1) an offline advertisement posted on bulletin boards around the Department of Psychiatry at Gangnam Severance Hospital and (2) a Web-based advertisement posted on a single website at Sungkyunkwan University, which has 2 campuses in South Korea. Volunteers who signed informed consent forms approved by the institutional review board (IRB) of Gangnam Severance Hospital, Yonsei University College of Medicine, were enrolled. None of the enrollees subsequently withdrew their participation. All of the enrolled participants had a normal or corrected-to-normal vision and no hearing or color-vision impairments, and none reported brain lesions or previous history of neurological or psychiatric disorders, including any current use of psychotropic medications. Out of 40 participants, 38 were right-handed (38/40, 95%), 1 was ambidextrous (1/40, 3%), and 1 was left-handed (1/40, 3%), as identified by the Edinburgh Handedness Inventory [25]. A total of 14 participants wore glasses, but those participants were required to adjust the VR glasses to their eye condition rather than wear their corrective glasses with the VR glasses. None of the participants reported a prior experience of enjoying 360° VR videos with the LG G5 smartphone-compatible VR glasses used in this study. The detailed demographic information on the enrolled participants is shown in Table 1.

**Table 1:** Demographic characteristics of the study sample (N=40).

Characteristics		Participants, n (%)
**Age (years)**		
	19-29	38 (95)
	30-39	2 (5)
**Gender**		
	Male	22 (55)
	Female	18 (45)
**Marital status**		
	Single	39 (98)
	Married	1 (3)
**Educational level**		
	Undergraduate	37 (93)
	Postgraduate	3 (8)
**Employment**		
	Unemployed	7 (18)
	Student	30 (75)
	Employed (full-time)	3 (8)
**Income**		
	Low	6 (15)
	Lower middle	15 (38)
	Upper middle	16 (40)
	High	3 (8)
**Smoking**		
	Nonsmoker	34 (85)
	Ex-smoker	3 (8)
	Smoker	3 (8)
**Alcohol drinking**		
	Never	9 (23)
	Once per week	18 (45)
	Twice per week	6 (15)
	3 times per week	5 (13)
	4 times per week	2 (5)
**Body mass index (kg/m^**2**^)**		
	Underweight (<18.50)	1 (3)
	Normal (18.50-24.99)	21 (53)
	Overweight (23.00-24.99)	11 (28)
	Obesity (25.00-29.99)	6 (15)
	Extreme obesity (≥30)	1 (3)

**Figure 2:**
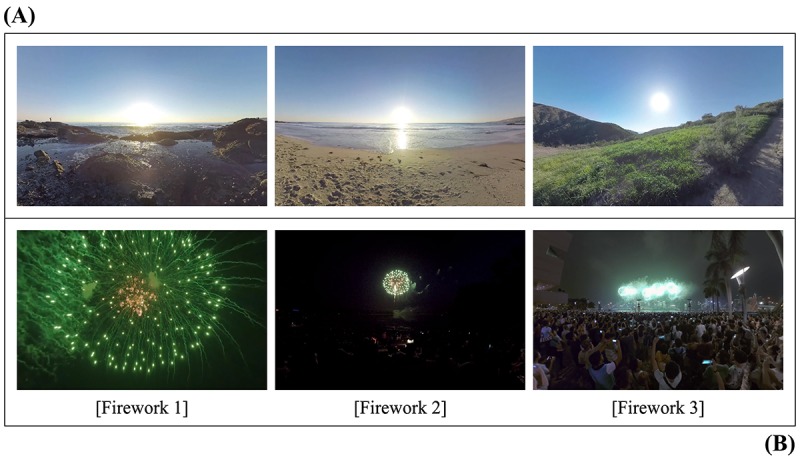
Screenshots of (A) three different types of hard-fascinating fireworks virtual environments (VEs) and (B) one mixed soft-fascinating nature virtual environment, including seaside, grassland, and hill locations.

### Visual Stimuli

As presented in Figure 2 the restorative nature 360° VE consisted of a variety of seaside, grassland, and hilly scenes. To confirm that the nature VE (with soft fascination) is more restorative than entertainment VEs (with hard fascination), 3 different fireworks VEs were collected (see Figure 2). Fireworks events, such as the annual Seoul International Fireworks festival held in October and the celebration on New Year’s Eve, are picturesque, attention-drawing entertainment events in city centers that Koreans and people around the world attend to drive away bad luck and evil spirits and to celebrate the New Year. The fireworks scenes were collected from Google’s YouTube. Because only the initial 5 min of a 10-min exposure to nature images was previously found to yield significant physiological responses [15], all of the 4K 360° VR videos were edited to the same running time of 5 min 53 s with the same resolution of 3840×1920 pixels.

### Apparatus

Auditory stimuli were delivered via MDR-1A headphones (Sony, Tokyo, Japan) on an OptiPlex 7040 Mini Tower PC (Dell, Round Rock, TX, USA) while the participants focused their attention on the given VR tasks. The experimental paradigm was programmed and presented using E-Prime v.2.0 software (Psychology Software Tools Inc; PST, Pittsburgh, PA, USA). Electroencephalographic (EEG) data were recorded with PST’s E-Prime Extensions for Net Station (EENS) v.2.0 and analyzed with the GES 400 system (Electrical Geodesics Inc; EGI, Eugene, OR, USA) using a Net Amps 400 amplifier, a 64-channel HydroCel Geodesic Sensor Net (HCGSN), and Net Station v.5.4 software (ie, Net Station Acquisition / Review / Tools) run by an Apple’s MacBook Pro (Apple Inc, Cupertino, CA, USA).

The mobile VR system (LG Electronics, Seoul, South Korea) consisted of an LG G5 smartphone and LG 360° VR glasses (960×720 pixels at 639 ppi per eye), which are only compatible with each other. In terms of comfort and wearability, the LG 360 VR glasses (164.1×185.6×45.9 mm; 134.3 g) were more appropriate in the experimental setting than the Samsung Gear VR released in 2015 (201.9×116.4×92.6 mm; 318 g, headset only), whose total weight was increased to approximately 480 g by the weight of the required smartphone. The LG VR glasses did not press down heavily on the electrodes placed around the forehead, eyes, and ears during pilot tests. For 360° VR videos to be optimally displayed on VR glasses across each participant’s vision, all participants were guided to manually adjust the focal length and inter-pupillary distance (the distance between the centers of the pupils of the 2 eyes). After calibrating the VR glasses, the participants were taught how to control the VR display and how to find, play, and view the 360° VR videos in the user interface.

### Experimental Paradigm and Visual Task

This ERP study employed a 2-tone passive auditory oddball paradigm in which frequent standard and infrequent deviant tones were presented with probabilities of 75% (180 trials) and 25% (60 trials), respectively. The 750 Hz standard tone and the 1000 Hz deviant tone were randomly presented at 75 dB sound pressure level for 100 ms, and the interstimulus interval varied randomly between 1000 ms and 1300 ms. Accordingly, the experimental paradigm was designed not to exceed a maximum of 5 min 30 s to prevent the VR stimuli from ending before the auditory oddball paradigm completely ended.

**Figure 3:**
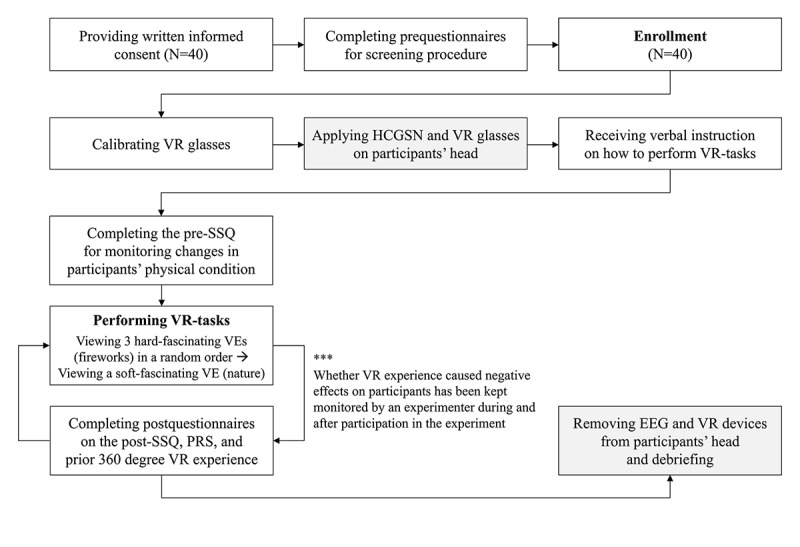
Flowchart of the experimental procedure. VR: virtual reality; HCGSN: HydroCel Geodesic Sensor Net; SSQ: Simulator Sickness Questionnaire; VE: virtual environment; PRS: Perceived Restorativeness Scale; EEG: electroencephalography.

While viewing silent 360° VR videos on a revolving chair, the participants were instructed not to intentionally detect sound changes or discriminate between the rare deviant tones and the frequent standard tones, which allowed them to focus only on the given visual task and ignore all the visual-task-irrelevant sounds and even any sudden noises in the EEG recording room. The 3 fireworks VEs were presented in a random order, followed by the single-nature VE. In accordance with the IRB-approved protocol presented in Figure 3, an experimenter constantly made sure that each time a VR video stimulus ended, the stimulus caused no inconvenience. Participants who showed any adverse reactions to the given stimuli would be withdrawn from the study as well as from the analyses. None of the enrolled participants reported problems with the EEG and mobile VR system or with the VR stimuli.

### Self-Report Measures

In the context of the 360° VR environment, it is important to determine whether the restorative experience in a nature VE could be threatened by simulator sickness. The Simulator Sickness Questionnaire (SSQ) [26] consists of a total of 16 items that measure the severity of 3 different groups of negative physical symptoms related to the experience of mechanical simulators: nausea (7 items: general discomfort, increased salivation, sweating, nausea, difficulty concentrating, stomach awareness, and burping), oculomotor (7 items: general discomfort, fatigue, headache, eye strain, difficulty focusing, difficulty concentrating, and blurred vision), and disorientation (7 items: difficulty focusing, nausea, fullness of head, blurred vision, dizzy [eye open and eye closed, respectively], and vertigo). Moreover, 3 subfactors of the SSQ contained overlapping symptom items. All items are rated from 0 (none) to 3 (severe). Following the recommendation of Kennedy et al [26], the SSQ was first administered to participants before they performed VR tasks to rule out the possibility of pre-existing symptoms and to measure the baseline physical condition (pre-exposure SSQ). The SSQ was subsequently administered immediately after viewing each 360° VE (postexposure SSQ). In this study, the Korean version of the SSQ was adopted from Min et al [27].

The current version of the PRS is composed of 26 items that measure the following 5 restorative factors: being away (5 items), fascination (8 items, including 2 reversed items), coherence (4 reversed items), compatibility (5 items), and legibility (4 items) [16]. The PRS was administered immediately after each VE to indicate how well certain statements described the participants’ restorative experience on a 7-point scale (0=not at all and 6=completely). To compute the total PRS score, the 6 reversed items were inversely coded, and then all the scores for the 5 restorative factors were summed. This study adopted the Korean version of the PRS scale from Yoo et al [28].

### Electrophysiological Data Recording and Analysis

During EEG recording, the analog signals were referenced to a single vertex electrode (Cz), filtered with a 0.01 to 400 Hz bandpass filter, and digitized at a sampling rate of 1000 samples per second online. The impedance for all wet electrodes was kept below 30 kΩ. The recommended threshold limit is 50 kΩ to ensure an optimal signal-to-noise ratio for the high-input impedance amplifier [29].

For preprocessing of the EEG signals, all datasets were refiltered with a 0.3 to 30 Hz bandpass filter offline and segmented into epochs ranging from 100 ms before to 500 ms after the onset of each of the 2 stimulus conditions: standard and deviant. On the basis of a timing test to ensure the accuracy of auditory stimulus presentation in E-Prime and EENS, the data were offset by the average offset value of 16 ms. Following the automated algorithm of EGI’s Net Station Tools, artifacts such as eye-blinks, eye-movements, and bad channels were detected. If a channel was bad for more than 20% of the segments, the channel was marked bad for all segments. Segments were marked bad if they contained (1) more than 10 bad channels (maximum-minimum>150 *μ* V for the entire segment, with a moving average of 80 ms), (2) eye-blinks (maximum-minimum>100 *μ* V, with a moving average of 80 ms), or (3) eye-movements (maximum-minimum>55 *μ* V, with a moving average of 80 ms). Bad channel replacement was then performed. The data were averaged for individual participants, and baseline corrected from −100 ms to 100 ms. Thereafter, a grand average was calculated using the data from all participants. After that, all of the channels were rereferenced to the average reference offline.

Finally, the deviant-standard difference waves were computed to identify the presence of the MMN/P3a complex component. For a further statistical analysis, the values of the adaptive mean of the aMMN and P3a amplitudes at the FCz electrode site were extracted from the different waveforms within the following time windows: aMMN: 150 to 250 ms; P3a: 220 to 280 ms.

## Results

### Manipulation Check

For a manipulation check, a one-way repeated measures analysis of variance (RM-ANOVA) was conducted to explore the effect of the type of VE (ie, soft or hard fascination) on the perception of environmental restorativeness. Before the RM-ANOVA, Mauchly test revealed that the assumption of sphericity had been met (Mauchly *W*=.79; *P*=.11; *ε*=.88). There was a significant effect of VE type (*F*_3,117_=27.18; *P*<.001; η*_p_*^2^=0.41). All 3 Bonferroni-adjusted post hoc comparisons of the PRS scores between the single nature VE and each of the 3 fireworks VEs indicated significant differences, which are nature (mean 101.25 [SE 3.91]) versus fireworks 1 (mean 69.97 [SE 3.57]), *P*<.001; nature versus fireworks 2 (mean 77.15 [SE 3.93]), *P*<.001; nature versus fireworks 3 (mean 81.08 [SE 3.85]), *P*<.001 (see Figure 2). The soft-fascinating nature VE was perceived as more restorative than the averaged hard-fascinating fireworks VE (mean 76.03 [SE 3.28]; *t*_39_=7.26; *P*<.001; Cohen *d*=0.78).

**Figure 4:**
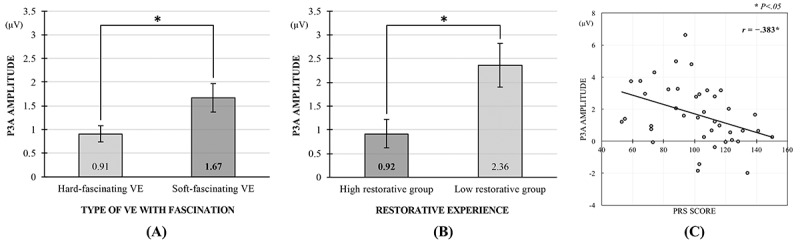
(A) Comparison of the P3a amplitudes while viewing hard-fascinating and soft-fascinating virtual environments. (B) Comparison of the P3a amplitudes for 2 groups with different levels of restorative experience from the soft-fascinating virtual environment. (C) Correlation between self-reported perceived restorativeness scores (PRS) and P3a amplitudes. VE: virtual environment. Asterisk indicates *P*<.05.

An ERP analysis of amplitude modulation in the MMN/P3a complex revealed that exposure to the 2 different VEs resulted in significantly different P3a amplitudes (*t*_39_=−2.05; *P*=.048; *d*=0.35) but not significantly different aMMN amplitudes (*t*_39_=0.59; *P*=.56). The increase in P3a amplitude elicited by unexpected and distracting stimuli was significantly greater during exposure to the soft-fascinating VE (mean 1.67 [SE 0.30]) than during exposure to the hard-fascinating VE (mean 0.91 [SE 0.17]; see Figure 4). However, there was no significant difference in the latency of the MMN/P3a complex between the 2 VEs (aMMN: *t*_39_=−1.23; *P*=.23 and P3a: *t*_39_=−0.61; *P*=.54).

In that respect, the nature VE stimuli were successful in eliciting participants’ restorative responses based on both subjective and neurophysiological measures. Furthermore, it was also evident that the aMMN might not depend on attention, and the amplitude of the MMN/P3a complex is more likely than the latency to reflect involuntary attention restoration.

### Simulator Sickness as a Control Variable

To clarify the genuine restorative effect of exposure to virtual nature, a one-way RM-ANOVA for the self-rating of simulator sickness before and after VR tasks was performed. As Mauchly test indicated that the assumption of sphericity had been violated (Mauchly *W*=.54; *P*=.006), the degrees of freedom were corrected using Greenhouse-Geisser estimates of sphericity (*ε*=.80). As no significant difference in all 5 SSQ scores was found (*F*_3.194,124.547_=2.42; *P*=.07), it was clear that the participants’ physical conditions did not vary with the VR exposure time and stimuli.

### Effect of Group Difference in Perceived Restorativeness on MMN/P3a Complex Modulation

To investigate how individuals’ restorative experience during exposure to virtual nature affected the MMN/P3a complex amplitudes, the 40 participants were divided on the basis of the median PRS score (103) into a low restorative group (PRS≤103, N=21; mean 82.62 [SE 3.65]) and a high restorative group (PRS>103, N=19; mean 121.84 [SE 2.88]).

The differences in the MMN/P3a complex amplitudes between the 2 restorative groups were evaluated using an independent samples *t* test. Whereas the difference in restorative experience failed to induce significantly different aMMN amplitudes (*t*_38_=0.91; *P*=.37), the high restorative group (mean 0.92 [SE 0.30]) showed significantly lower P3a amplitudes than the low restorative group (mean 2.36 [SE 0.46]) when viewing the soft-fascinating nature VE (*t*_38_=2.57; *P*=.02; *d*=0.59; see Figure 4). Consistent with the result of the manipulation check, the latency of the MMN/P3a complex did not significantly differ between the 2 groups (aMMN: *t*_38_=−1.4;, *P*=.15 and P3a: *t*_38_=−0.31; *P*=.76).

**Figure 5:**
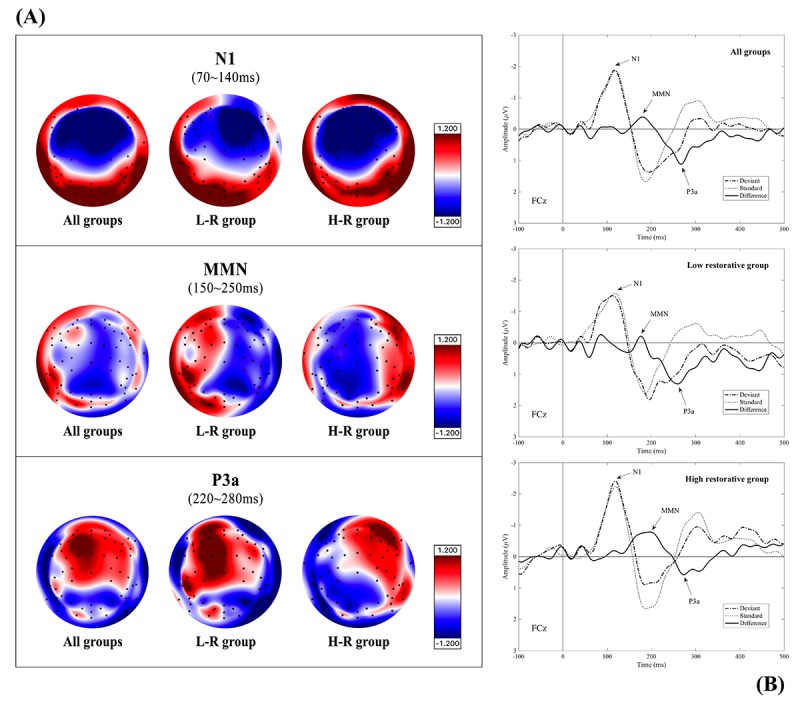
(A) Event-related potential topographic maps of the N1 for deviant tones and the MMN/P3a complex for the deviant-standard difference wave. (B) Time courses of the N1 and mismatch negativity/P3a complex components for FCz, which were shown by each of the 2 restorative groups during exposure to virtual nature. L-R group: low-restorative group; H-R group: high restorative group.

As the ERP reflection of normal cognitive functioning and involuntary attention restoration, the fronto-central N1/aMMN and P3a components were prominently produced by deviant tone detection while viewing 360° virtual nature. In particular, the high restorative group exhibited more attenuated fronto-central P3a activity in the topographical distributions than the low restorative group, which is consistent with decreased involvement of the frontal cortex in response to auditory distractors during meditation (see Figure 5). Figure 5 shows the ERP time courses of the 2 groups with different levels of restorative experience at the FCz electrode site.

### Correlations Between Individual’s Perceived Restorativeness Scale Scores and P3a Amplitudes

A correlation analysis was conducted to examine the relationship between self-reported PRS scores and P3a amplitudes over the 3 fronto-central midline electrode sites: Fz, FCz, and Cz. Following the interpretation of Cohen guideline [30], there was a moderate but significant negative correlation between the explicit and implicit measures at the FCz site (*r*=−.38; *P*=.02; see Figure 4) but not at the Fz (*r*=−.11; *P*=.50) and Cz (*r*=−.28; *P*=.08) sites. A significantly negative correlation between the PRS score and the P3a amplitude at the FCz electrode is in line with the decreased amplitude of the P3a after meditation practice, which may indicate decreased attentional engagement by the frontal cortex [21,22]. However, the aforementioned studies collected limited EEG data at only the Fz and Cz sites by using a low-density 19-channel electrode cap compared with the 3 electrode sites incorporated in the high-density 64 channel sensor net used in this study. In that context, the result reflects the P3a characteristic as an auditory ERP component measured mainly at the fronto-central region (ie, FCz or Cz).

## Discussion

### Principal Findings

The aim of this ART-based study is to determine whether the amplitude of the MMN/P3a complex can be a distinct ERP biomarker of involuntary attention restoration during exposure to 360° virtual nature in healthy young adults with different levels of restorative experience. Unlike previous studies that look at aMMN or P3a separately, this study focused on the neurophysiological handover from preattentive information processing (ie, aMMN) to subsequent attention orienting processing (ie, P3a) as indexed by the MMN/P3a complex. The findings of previous ART and ERP studies in the real world extend into those of this study in the virtual world. The theoretical and empirical implications of this study will support the development and assessment of restorative VR content by allowing various stakeholders such as designers, researchers, and clinicians to investigate the restorative responses that potential users have to active relaxation in restorative VEs, particularly at the level of involuntary attention (previous studies were limited to voluntary attention).

As shown by the manipulation check and the hypothesis testing, there were no significant differences in the aMMN amplitudes between the 2 restorative groups or between the 2 fascinating VE conditions, which corresponds to the preattentive nature of the aMMN, beyond the attention debate in the real world [24], and the condition that participants had to focus only on conducting visual tasks and could ignore any environmental sounds. In other words, not only is automatic change detection one of the most important cognitive functions for human survival from an evolutionary perspective but also the normal functioning of the cognitive system is characterized by maintaining a sound balance between goal-directed behavior and involuntary orientation [31,32]. Despite no significant changes in the aMMN amplitudes during exposure to virtual nature, the robustness of the aMMN amplitudes indicates that the study participants qualified as healthy subjects. Mental fatigue during or after prolonged periods of cognitive tasks impairs preattentive auditory processing, as revealed by the fact that aMMN amplitudes at fronto-central electrode sites can be significantly decreased by mental fatigue [33]. Such impairments in the preattentive processing of auditory change detection also appear in clinical populations, such as patients with schizophrenia [34,35], attention deficit disorder or attention-deficit hyperactivity disorder [36], or major depressive disorder [37]. Given the possible link between decreased exposure to nature and increased occurrence of mental disorders [6-8], it is important to clarify the role of the aMMN amplitude in different groups of people, including those who are vulnerable to mental fatigue, at high risk for mental disorders, or currently suffering from acute or chronic mental disorders.

On the basis of the few ART studies that focused on the concept of fascination [1,5,38], this study compared the restorative effect of entertaining events with that of natural events for the manipulation check. Consistent with the ART [1], the less restorative urban fireworks VEs with hard fascination consumed relatively more involuntary attentional resources and left little space for mental reflection compared with the more restorative nature VEs with soft fascination, which elicited significantly increased P3a amplitudes and PRS scores. As hypothesized, the P3a amplitude was differently affected by individuals’ restorative experience during exposure to the nature VE, supporting the restorative effect of virtual nature as indicated by the negative correlation between self-reported PRS scores and P3a amplitudes as well as by the attenuated P3a amplitudes in the high restorative group. As the P3a is an index for frontal neural activity generated by stimulus-driven attention mechanisms [22], a trend for clear P3a amplitude reduction is consistent with decreased attentional engagement by the frontal cortex in response to unexpected and distracting stimuli during meditation [21]. In line with the finding of Pierson et al [39], it is more likely that participants who report greater restorative depth find it easier to enter a state of deep quiescence because of their low sensation-seeking trait, resulting in a positive correlation between restorative depth and frontal P3a amplitude. More importantly, meditation not only reflects first-person experiences that cannot be easily shared [40] but also facilitates attention control mechanisms [41,42]. If the restorative effect of virtual nature experience shows a trend similar to that of meditation practice with the aim of decreasing emotional and cognitive reactivity, individuals’ restorative experience in a nature VE could play a key role in renewing the capacity for attention control without an intentional effort to regulate attention [1,4]. To shed light on the similarities between the 2 relaxation techniques, future studies need to determine whether the decreased automated reactivity and inhibited evaluative processing related to the task-irrelevant, attention-demanding auditory stimuli are consistent phenomena in other groups of individuals.

This study has some limitations. Although meditation is well known as an effective relaxation technique with both short-term and long-term effects on attentional function [21,43-45], only the short-term effect of exposure to 360° VR nature were examined by collecting normative data from a relatively small sample with a narrow age range and strict exclusion criteria. To generalize the findings, the restorative effect of short-term and long-term exposure to nature VR in people with neuro or psychiatric disorders as well as in younger or elderly adults need to be examined. Mental and attentional fatigue might make it difficult to meditate without guidance or help from professionals. However, some people might want to meditate for long periods of time or in a flexible manner, so they prefer nonguided meditation or free-style relaxation to guided meditation. For those reasons, a self-chosen 360° VR nature therapy within a short time might be more appealing than a virtually guided scenario-based VR meditation. The mobile VR system might allow mentally fatigued workers and students to feel some relief from real-world stressful factors as if they had actually experienced a period of vacation. In addition, people with disabilities, elderly people, and hospital patients would be able to easily use VR nature therapy in the home and ward environments. Individuals who are vulnerable to simulator sickness could control the 360° VEs with subtle head movements or even without wearing VR glasses and, thus, could act independently in the virtual world. Taken together, the results of this study suggest that VR nature therapy may one day provide a self-help, self-administered treatment for mental and attentional fatigue in everyday life.

### Conclusions

The 360° VR nature exposure therapy can help individuals suffering from mental fatigue to manage their attentional resources with minimum effort. About 5 min of exposure to virtual nature restored involuntary attention without causing simulator sickness. The amplitude of the fronto-central MMN/P3a complex can potentially be employed as a distinct ERP biomarker of involuntary attention restoration during virtual nature experience in healthy young adults.

## Abbreviations

aMMN: auditory mismatch negativity
ART: attention restoration theory
EEG: electroencephalography
EENS: Extensions for Net Station
EGI: Electrical Geodesics Inc
ERP: event-related potential
HCGSN: HydroCel Geodesic Sensor Net
IRB: institutional review board
MMN: mismatch negativity
PRS: Perceived Restorativeness Scale
PST: Psychology Software Tools Inc
RM-ANOVA: repeated measures analysis of variance
SSQ: Simulator Sickness Questionnaire
VE: virtual environment
VR: virtual reality
